# Practice and outcomes of neonatal resuscitation for newborns with birth asphyxia at Kakamega County General Hospital, Kenya: a direct observation study

**DOI:** 10.1186/s12887-018-1127-6

**Published:** 2018-05-15

**Authors:** Duncan N. Shikuku, Benson Milimo, Elizabeth Ayebare, Peter Gisore, Gorrette Nalwadda

**Affiliations:** 1 Department of Nursing, Makerere University, School of Health Sciences, Kampala, Uganda; 2 Department of Midwifery and Gender, Moi University, School of Nursing, Eldoret, Kenya; 3 Department of Child Health & Pediatrics, Moi University, School of Medicine, Eldoret, Kenya

**Keywords:** Birth asphyxia, Neonatal resuscitation, Newborn, Kenya

## Abstract

**Background:**

About three – quarters of all neonatal deaths occur during the first week of life, with over half of these occurring within the first 24 h after birth. The first minutes after birth are critical to reducing neonatal mortality. Successful neonatal resuscitation (NR) has the potential to prevent these perinatal mortalities related to birth asphyxia. This study described the practice of NR and outcomes of newborns with birth asphyxia in a busy referral hospital.

**Methods:**

Direct observations of 138 NRs by 28 healthcare providers (HCPs) were conducted using a predetermined checklist adapted from the national pediatric resuscitation protocol. Descriptive statistics were computed and chi – square tests were used to test associations between the newborn outcome at 1 h and the NR processes for the observed newborns. Logistic regression models assessed the relationship between the survival status at 1 h versus the NR processes and newborn characteristics.

**Results:**

Nurses performed 72.5% of the NRs. A warm environment was maintained in 71% of the resuscitations. Airway was checked for almost all newborns (98%) who did not initiate spontaneous breathing after stimulation. However, only 40% of newborns were correctly cared for in case of meconium presence in airway. Bag and mask ventilation (BMV) was initiated in 100% of newborns who did not respond to stimulation and airway maintenance. About 86.2% of resuscitated newborns survived after 1 h. Removing wet cloth (*P* = 0.035, OR = 2.90, CI = 1.08–7.76), keeping baby warm (*P* = 0.018, OR = 3.30, CI = 1.22–8.88), meconium in airway (*P* = 0.042, OR = 0.34, CI = 0.12–0.96) and gestation age (*P* = 0.007, OR = 1.38, CI = 1.10–1.75) were associated with newborn outcome at 1 h.

**Conclusions:**

Mentorship and regular cost – effective NR trainings with focus on maintaining the warm chain during NR, airway maintenance in meconium presence, BMV and care for premature babies are needed for HCPs providing NR.

**Electronic supplementary material:**

The online version of this article (10.1186/s12887-018-1127-6) contains supplementary material, which is available to authorized users.

## Background

Globally, approximately 4 million deaths occur in neonates with 99% of them occurring in low and middle income countries [1]. Birth asphyxia, defined as the failure to initiate and sustain breathing at birth by WHO, causes about a quarter of all the neonatal deaths [2]. Approximately three – quarters of all neonatal deaths occur during the first week of life, with a million babies dying on the day they are born [3]. Conspicuously, over half of these neonatal deaths occur within the first 24 h after birth [4, 5]. However, morbidity and mortality from birth asphyxia is mostly preventable and treatable [6]. Effective resuscitation at birth can prevent a large proportion – approximately 30% - of these deaths [7]. Furthermore, resuscitation may avert 5–10% of deaths due to complications of preterm birth [8].

Evidence around the world also show that the risk of death increases by 16% for every 30 s delay in initiating ventilation up to six minutes and every 6% for every minute of delay of applied bag and mask ventilation [5]. Therefore, it is clear that the first minutes after birth are critical to reducing neonatal mortality. Evidence suggests that successful neonatal resuscitation by well – trained HCPs to provide appropriate and adequate resuscitation has the potential to prevent perinatal mortality caused by intrapartum related asphyxia for almost two million babies annually [3]. Newborns with birth asphyxia can suffer from short- to long-term neurological complications. Severe asphyxia has been linked to cerebral palsy, mental retardation, epilepsy and learning disabilities [9]. Therefore, there is need for urgent referral of complicated birth asphyxia cases to higher well specialized facilities in order to assist prevent some of these complications if anticipated early enough [6].

Neonatal mortality in Kenya is still high at 21 per 1000 live births [10] with Kenya aiming to achieve the global SDG target of neonatal mortality to at least as low as 12 per 1000 live births by 2030 [11]. Birth asphyxia is the leading cause of neonatal mortality contributing to 29% of the deaths in the country [12]. The ministry of health (Kenya) recognizes the importance of neonatal resuscitation (NR) as part of the basic emergency obstetric and newborn care (BEmONC) from level 2 health facilities [13]. National guidelines in the form of protocols have been developed and reviewed over time to standardize the performance of NR for newborns with birth asphyxia [14].

However, despite the HCPs’ pre – service and in – service training, guidelines and job aids with adequate equipment in NR, practices of HCPs with regard to NR are still reported to be poor [15–17]. Ineffective or wrong resuscitation practices are linked to the persistently high neonatal deaths from birth asphyxia in the first 1–24 h [18]. Kakamega county region is the second most populated region in the country with a high neonatal mortality rate of 28 per 1000 live births [19]. To better describe the healthcare practice and outcomes, the three Donabedian domains of healthcare assessment: structures/resources, process and outcomes are used [20]. This study aimed to describe the practice of NR and outcomes of newborns with birth asphyxia in a busy main regional referral hospital.

## Methods

This was a cross - sectional study employing direct observations of NR in labour ward and maternity theatre between April and June 2016 at the regional Kakamega County General Hospital. This method is non – intrusive, where the HCPs do what they normally do (resuscitation) without being interrupted or disturbed by the observer [21, 22]. This allowed the HCPs to be observed in their natural fashion providing NR without interfering in their process of care. However, the newborn unit was excluded as newborns are only referred here after stabilizing from the initial resuscitation done in labor ward or maternity theatre immediately after delivery.

All the HCPs who were involved in resuscitating a newborn and voluntarily consented to participate in the study were observed during resuscitation sessions. The HCPs should have been working in the labor ward and/or the maternity theatre and providing direct NR services. Trainee nursing and medical students were excluded (unlicensed to practice). Newborns with birth asphyxia who met the inclusion criteria based on the WHO and the American Academy of Pediatrics were recruited [2, 23]. The criteria included: failure to initiate spontaneous respirations at birth/within 1 min of delivery and/or gasping breathing at 30 s after birth and/or baby is floppy and/or bluish or has central cyanosis (blue tongue). Stillbirths (birth of a baby who shows no signs of life [no gasping, breathing, heartbeat or movement]) and those with congenital abnormalities incompatible with life were excluded. Consecutive sampling was used to select all the newborns that required NR immediately after birth and met the inclusion criteria until the required sample size was achieved. It is estimated that about 1 in 10 babies needs help to breathe immediately after birth and therefore, a quick assessment immediately after birth remains the best way to know if a baby needs help to breathe [24]. The Kish Leslie formula (**n = Z**^**2**^ **pq/e**^**2**^**)** for cross – sectional studies was used to calculate the sample size of the NRs to be observed [25]. The **Z** (variate from normal distribution that represents the level of confidence) was 1.96; **p** (estimated proportion of attribute present in a population) was set at 10% as the number of newborns who require resuscitation to breathe at birth [24]; and **q** = 1 – p. The desired level of precision **(e)** was set at 95% (minimum acceptable errors at 5%) giving a total of 138 as the desired newborn resuscitations to be observed. Each HCP was observed for between three to five times providing NR care.

### Study procedures

The hospital’s labor ward and theatre had a common central resuscitation area where all NRs were performed. Four research assistants (RAs) (one for day shift, one for evening shift and two for night shifts) were recruited and observed the NRs. The RAs were nurses recruited from among the hospital nurses from the antenatal ward with experience and formal NR training. This was to minimize the Hawthorne effect associated with observation studies [26]. This was on the assumption that the HCPs were less likely to change their practices when being observed by another HCP in the same unit as opposed to an observer from outside the hospital. Thus, they were not given any formal NR training by the primary investigator but were given a two day instruction/training on how to observe resuscitations against a predetermined checklist. Two practical observations were done with the principal investigator to ensure that all the RAs used the checklist in a similar manner.

#### Preparation phase

Significance of the study was explained to HCPs and written informed consent from HCPs was sought with emphasis on voluntary participation. The HCPs were sensitized once on the researcher’s intent to observe the actual resuscitation without subsequent reminders during the actual procedure. Upon consenting, HCP’s biodata details were completed in the structured direct observation checklist.

#### Data collection

All the three shifts (morning, evening and night duty) were covered in order not to miss out on the resuscitations. A research assistant was present at the resuscitation area every time a delivery was being conducted either in theatre or delivery room. In case of two resuscitations occurring at the same time, the observer went to the delivery that began first. However, this situation only occurred twice. The observer strategically positioned herself near the resuscitaire in order to have a clear view of the resuscitation process from the start to end.

First, information was collected about the availability, functionality and accessibility of the essential NR equipment at the resuscitaire. Once a newborn was delivered, the observer included the resuscitation only if two criteria were fulfilled. First, the HCP receiving the newborn had consented to participate in the study and the newborn delivered required resuscitation (met the eligibility criteria). The RA observed the actual NR as conducted by the HCP on the ward against the predetermined checklist to assess the skills of the HCP under observation (See structured observation checklist in Additional file 1).

The assessment of the skill focused on the following areas: preparation, the Airway, Breathing and Circulation. Being a direct observation study, the researchers tried their best not interfere in the NR process as it is non – intrusive [21, 22]. The study focused on the HCPs NR techniques as routinely done by HCPs individually. However, in case of practices deemed to be harmful or compromising resuscitation and likely to lead to irreparable damage/death of the neonate, the observer assisted by calling for help from the other HCP to intervene so as to ensure that the neonate would be given the greatest chance of survival as recommended [2, 24]. The RAs then recorded any inappropriate or harmful NR practices observed including holding the neonate upside down, shaking the neonate vigorously, hard patting/slapping of neonate on back, flicking foot of the neonate, vigorously wiping neonate and squeezing chest of neonate.

At the end of the NR, infection prevention practices were observed. This included decontamination and disinfection of the resuscitation equipment such as suction device, mask and oxygen tubing for next use.

Stepwise immediate and at 5 min, 10 min and 1 h outcomes for the resuscitated babies were monitored and documented. This included outcomes after drying/stimulation, clearing of airway and adequate bag and mask ventilation. Other outcomes e.g. death were captured indirectly under the “others” since they may be attributed to other factors (confounders) not directly related to NR. This helped us assess the specific intervention for each newborn resuscitated differently during the resuscitation process based on the outcome at each step of resuscitation undertaken.

#### Data quality control and management

The structured observation checklist was pretested first at the Mulago Hospital labor ward of Uganda to ensure reliability and validity in the data collected. Random visits by primary investigator were conducted to ensure that observations were being carried out as instructed and checklists were being filled in on site. At the end of the shifts, forms and checklists were checked by the primary investigator for completeness and errors before leaving the study area/site. The data collected was be kept strictly confidential.

#### Variables and measurements

The primary outcome was the newborn’s outcome status at 1 h. This was a binary outcome: alive or dead. The independent variables influencing the newborn outcome focused on the Donabedian domains of structures and processes. The independent process variables expected to influence the newborn outcome were also constructed as binary with “yes = 1” and “no = 0.” Process variables/items were broadly classified under the 3 principle areas of NR process. They were: drying/stimulation [three items], airway maintenance [four items], bag and mask ventilation [seven items] including the optional cardiopulmonary resuscitation [two items] (Table 3). Structural/input factors were measured by NR equipment availability, HCP cadre, training/qualifications and experience, support staff supervision and NR trainings.

Data were captured using the Microsoft Office Excel 2013 software and exported to STATA version 13 for analysis. The unit of analysis was the resuscitated neonate. All the data from HCPs who had 3–5 observations were analyzed. This was a representative and appropriate comparison that eliminated the early initial fears of the HCP of being observed during the practice [26].

For process indicators, descriptive statistics were used and where proportions were calculated, 95%CI were computed. Pearson’s chi – square tests were used to test the associations between the newborn outcome at 1 h and the NR processes and newborn characteristics. Logistic regression models were used to assess the association between the process indicators (NR steps) and the primary outcome (survival at 1 h). However, since the NR steps are dependent, the regression was done independently for each NR step performed. Newborn characteristics that were significant in the bivariate analysis were retained as predictors in the final model. With a 95% confidence interval, *P* – values ≤0.05 were considered statistically significant.

## Results

### Health care providers background characteristics

Twenty eight HCPs with a median age of 30 years (range 24–50) participated in the study. Nurses/midwives were the majority cadre (71.4%) providing newborn resuscitation. Two thirds of nurses/midwives (65%) were registered diploma holders and one third (35%) were bachelor degree nurses. Most of the HCPs (89.3%) had worked in the maternity ward providing NR care for more than a year. Eighteen HCPs (64.3%) reported ever attending a NR training. Most HCPs (66.7%) had such trainings over 12 months prior to this study. Importantly, all the HCPs who participated in the study had at least undergone either a formal NR training or a non – specific NR induction training (the initial orientation offered to new staffs joining the maternity unit on a number of emergency obstetrics and neonatal care skills e.g. neonatal resuscitation, manual removal of the placenta, maternal resuscitation etc. at the hospital). More than half (*n* = 17, 60.7%) of the HCPs had attended the Basic Emergency Obstetrics and Neonatal Care (BEmONC) training that has a component on NR with at least half (*n* = 9, 52.9%) of them completing the training within the past six months prior to the study. Support supervision in NR was reported by most HCPs (89.3%). Over half of the HCPs (60%) reported having received the supervision within the past year prior to this study by the maternity unit manager and/or the labour ward in – charge.

Nurses provided NR for the majority of the newborn babies (72.5%). Majority of the newborn babies (63.8%) were cared for by HCPs who had undergone a NR training. The HCPs with over a year experience working in maternity performed most of the NRs (89.1%) (See Table 1).

**Table 1:** Background characteristics of HCPs with the distribution of newborns resuscitated at KCGH

HCP Characteristics	Frequency (*n* = 28)	Percentage (%)	Newborns resuscitated (*n* = 138)	Percentage (%)
Age (years)				
< 25 years	3	10.7	15	10.9
> 25–50 years	25	89.3	123	89.1
Sex				
Male	8	28.6	38	27.5
Female	20	71.4	100	72.5
Professional Cadre				
Nurses/midwives	20	71.4	100	72.5
Medical officers	4	14.3	20	14.5
Anesthetists	3	10.7	13	9.4
Clinical officers	1	3.6	5	3.6
Qualification				
Diploma	15	53.6	73	52.9
Bachelor degree	13	46.4	65	47.1
Previous training in NR				
Yes	18	64.3	88	63.8
No	10	35.7	50	36.2
Period since the last NR training (*n* = 18)^a^			(*n* = 88)	
< 6 months	6	33.3	30	34.1
≥ Over 12 months	12	66.7	58	65.9
Support supervision in NR				
Yes	25	89.3	123	89.1
No	3	10.7	15	10.9
Period since last support supervision in NR (*n* = 25)			(n = 123)	
Past 6 months	9	36.0	45	32.6
> 6–12 months	6	24.0	30	21.7
> 12 months	10	40.0	48	34.8
Period working in maternity				
< 1 year	3	10.7	15	10.9
> 1 year – 5 years	17	60.7	83	60.1
≥ 5 years	8	28.6	40	29.0

^a^no HCP trained within last 6–12 months

### Health facility characteristics

Helping Babies Breathe (HBB) NR action plans and guidelines were displayed at the resuscitation area. No immediate newborn care and warm chain charts were observed in the unit. All the basic NR equipment: two resuscitaires equipped with electric warmers; oxygen source (two oxygen cylinders, oxygen flow meters and oxygen tubing); suction devices (electric suction machine & colored suction bulbs); ambubags; term face masks (size 1) and the wall clock were available, functional and accessible at the common resuscitation station. However, only one functional preterm mask (size 0) was available and occasionally shareable between the labor ward and the newborn unit. A clean dry towel for drying the newborn was present in each delivery pack.

### Infection prevention practices

In 97.8% (*n* = 135) resuscitation cases, equipment used (suction devices and face masks) were well processed with the HCPs adhering to recommended infection prevention practices. These were cleaning of ventilating face mask and the suction device, decontamination in 0.5% sodium hypochlorite solution for 10 min (although occasionally some stayed longer than the 10 min), washing with soap and water and rinsed well and aired to dry until next use.

### Processes of care

#### General characteristics of newborns resuscitated

A total of 1569 deliveries were conducted at the hospital during the study period. Observations of 138 NRs performed by 28 HCPs were done by the RAs. Majority of the deliveries (*n* = 82, 59.4%) were by spontaneous vertex delivery (SVD) and cesarean section (*n* = 49, 35.5%). The mean birth weight was 3211.2 g (SD ± 936.0); lowest had 900 g and highest had 5000 g. Thirty (21.7%) newborns had low birth weight (< 2500 g) while 51 (37.0%) of the newborns were preterm births with gestational ages less than 37 weeks. Fifty seven (41.3%) newborns had meconium present in their airway at delivery (See Table 2).

**Table 2:** Characteristics of the resuscitated newborns

Characteristic	Frequency (*n* = 138)	Percentage (%)
Mode of delivery		
SVD	82	59.4
C/S	49	35.5
Breech extraction	6	4.4
Others^a^	1	0.7
Birth weight (grams)		
< 2500 g	30	21.7
≥ 2500 g	108	78.3
Gestational age (weeks)		
< 37 weeks	51	37.0
≥ 37 weeks	87	63.0
Meconium present		
Yes	57	41.3
No	81	58.7

^a^Others included assisted delivery by vacuum extraction

#### Overall neonatal resuscitation processes performed

Nearly all (*n* = 122, 88%) the newborns were dried gently by rubbing the back with the dry towel. However, the wet towel was not removed in a third of the newborns (31%) resuscitated. A few inappropriate stimulation practices observed during the resuscitations included: vigorously rubbing the baby’s back and chest (*n* = 11, 8%), flicking the baby’s feet (*n* = 3, 2.2%) and patting the baby’s back (*n* = 1, 0.7%).

The airway was checked and cleared for all (*n* = 123) newborns who did not respond to stimulation with any form of airway secretions. Meconium was present in the airway in 57 (46%) of the newborns. Suctioning of airway before stimulation in presence of meconium as per the national guidelines was only correctly done in 23 (40%) of the newborns. Inappropriate head positioning (head not in neutral position to facilitate airway opening) to clear the airway was observed in 21 (17%) cases and 11 (7.2%) of the newborns were turned upside down and back patted. A few (*n* = 6, 4.9%) newborns required prolonged suctioning with a bulb suction device for over 10 min to open the airway.

Bag and mask ventilation (BMV) was initiated for all the newborns who did not initiate breathing after airway clearance (*n* = 66, 100%). Ventilation was initiated within the Golden minute in just over half (*n* = 36, 54.6%) of the newborns who required help. The mean time for initiation of bag valve and mask ventilation was 69.2 s (SD ± 19.6). Less than half (*n* = 30, 45.5%) of the newborns did not respond after the initial BMV for a minute and required advanced/subsequent BMV. Chest compressions with effective breaths and supplemental oxygen were provided for the newborns who did not initiate spontaneous breathing after the advanced BMV as per the national guidelines (See Table 3). Outside the national guidelines, administration of intravenous 10% dextrose through the newborn’s umbilical vein was observed in nine (6.5%) cases who needed ventilation support.

**Table 3:** Neonatal resuscitation performance at the Kakamega County General Hospital

NR step performance	Number	Percent	95% CI
Drying/Stimulation (*n* = 138)			
Baby dried thoroughly by gently rubbing the back	122	88	83–94
Wet cloth removed	95	69	61–77
Baby kept warm	98	71	63–79
Airway clearance (*n* = 123)			
Checked airway	121	98	96–100
Meconium ©	57	46	37–55
If meconium, suctioning done before stimulation	23	40	27–53
Airway cleared with suction bulb if unresponsive	123	100	
Baby’s head in neutral position	102	83	76–90
Bag and mask ventilation for breathing (*n* = 66)			
Initial BMV initiated within the Golden minute	36	55	42–67
Subsequent BMV (n = 30)			
HCP call for help	26	87	74–100
Correct mask size used during BMV	25	83	69–97
Chest movements observed with each ventilation	19	63	45–82
BMV rate within 30–50 breaths/minute	18	60	41–79
Baby’s HR checked at 1 min	22	73	56–90
Cardiopulmonary resuscitation (*n* = 24)			
Chest compressions	20	83	67–99
Supportive oxygen	21	88	73–102

*Meconium* © meconium present in airway, *BMV* Bag and mask ventilation, *HCP* Healthcare provider, *HR* Heart rate). Percentages rounded off to the nearest whole number

### Neonatal outcomes

#### General neonatal outcomes after resuscitation

All the newborns (*n* = 138, 100%) resuscitated were followed up from 1, 5, 10 min and at 1 h. The mean APGAR scores were 5.9 (SD ±1.3) at 1 min, 6.9 (SD ± 2.0) at 5 min and 8.1 (SD ± 2.9) at 10 min for the 138 newborns resuscitated. More than half (*n* = 73, 52.9%) of the newborns had APGAR scores less than 6 at 1 min with the remainder (*n* = 65, 47.1%) having APGAR scores of 7 at 1 min. Overall, majority of the neonates (*n* = 119, 86.2%) survived. Of the 19 (13.8%) who died, a third (n = 6, 31.6%) died before 10 min after birth with the remaining deaths (*n* = 13, 68.4%) occurring within the first hour of life.

**Fig. 1:**
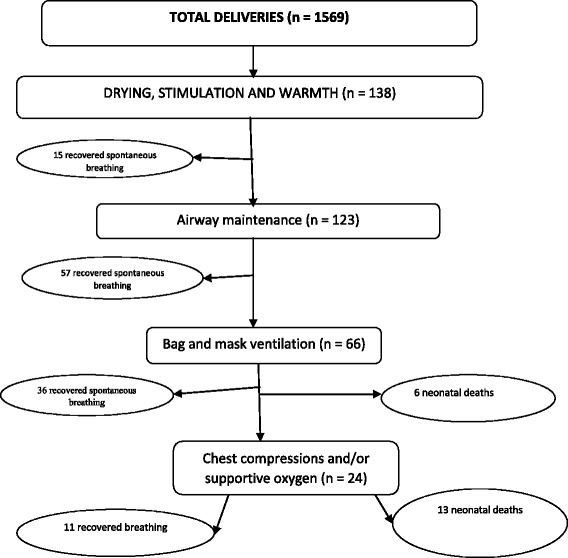
Neonatal outcomes after specific resuscitation treatment

Over half (*n* = 72, 52.2%) of the newborns initiated spontaneous breathing after the initial drying and airway suction stimulation. Less than half (*n* = 66, 47.8%) of the newborns required bag and mask ventilation to initiate spontaneous breathing (See Fig. 1).

#### Type of care post neonatal resuscitation at 1 h

**Fig. 2:**
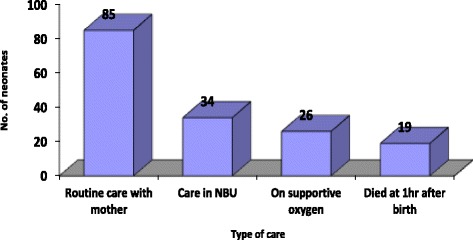
Type of care post neonatal resuscitation at 1 h

Of the neonates who were breathing at 1 h, 85 (71.4%) were under normal routine care with the mother and the remaining 34 (28.6%) were in special newborn care unit for supportive oxygen and/or other specific management for the newborns at risk after birth (See Fig. 2). Unstable newborns after the standard bag and mask ventilation with room air were referred to the facility’s newborn unit for positive pressure ventilation – oxygen administration via the nasal cannulae and intravenous fluids administration. Other care included antibiotics administration, nasogastric tube feeding and kangaroo mother care as per the national guidelines on essential newborn care. Newborns requiring specialized positive pressure ventilation - Continuous Positive Airway Pressure (CPAP) are usually referred to the nearby national teaching and referral hospital’s newborn intensive care unit. No newborns were transferred for this specialized care during the period.

### Association between the neonatal resuscitation practices and neonatal outcomes

Pearson’s chi – square tests for removing wet cloth (X^2^ = 4.7359, *P* = 0.030), keeping the baby warm (X^2^ = 5.9852, *P* = 0.014), meconium presence in airway (X^2^ = 4.4055, *P* = 0.036), suctioning before stimulation in meconium presence (X^2^ = 4.3613, *P* = 0.037) and chest movements with each bag and mask ventilation (X^2^ = 4.6355, *P* = 0.031) showed statistically significant associations with the neonate’s survival at 1 h. In addition, neonate’s gestation age (X^2^ = 12.76, *P* < 0.001) and birth weight (X^2^ = 16.93, *P* = 0.001) were significantly associated with the neonate’s survival or death (See Table 4).

**Table 4:** Association between neonatal resuscitation processes, newborn characteristics and neonatal outcomes

NR process		Neonate’s Outcome at 1 h., n (%)		X^2^	*P* value
		Alive	Died		
Baby dried by gently rubbing the back		105 (86%)	17 (14%)	0.0245	0.876
Wet cloth removed		86 (90.5%)	9 (9.5%)	4.7359	0.030*
Baby kept warm		89 (90.8%)	9 (9.2%)	5.9852	0.014*
Looked into airway		103 (85.1%)	18 (14.9%)	1.8584	0.173
Meconium		44 (77.2%)	13 (22.8%)	4.4055	0.036*
Suctioning before drying in meconium		21 (91.3%)	2 (8.7%)	4.3613	0.037*
Head in neutral position		89 (87.2%)	13 (12.8%)	3.3396	**0.068**
BMV within Golden minute		26 (72.2%)	10 (27.8%)	0.0394	0.843
Call for help		44 (73.3%)	16 (26.7%)	1.4486	0.229
Correct mask size during BMV		43 (72.9%)	16 (27.1%)	0.7561	0.385
Chest movements with BMV		39 (78.0%)	11 (22.0%)	4.6355	0.031*
BMV within 30 - 50b/min		34 (75.6%)	11 (24.4%)	1.3015	0.254
HR checked		38 (74.5%)	13 (25.5%)	1.1904	0.275
Effective breath/chest compressions		8 (40.0%)	12 (60.0%)	1.6448	0.200
Supportive oxygen		11 (52.4%)	10 (47.6%)	2.9011	**0.089**
Newborn characteristics					
Gestation age (weeks)	**< 37**	37 (72.6%)	14 (27.5%)		
	≥37	82 (94.3%)	5 (5.8%)	12.76	< 0.001*
Birth weight	< 2500 g	19 (63.3%)	11 (36.7%)		
	≥2500 g	100 (92.6%)	8 (7.4%)	16.93	0.001*
Mode of delivery	SVD	68 (82.9%)	14 (17.1%)		
	C/S	44 (89.8%)	5 (10.2%)		
	Breech	6 (100%)	0 (0%)		
	Others	1 (100%)	0 (0%)	2.40	0.494

*P ≤ 0.05 statistically significant

### Relationship between the newborn resuscitation processes and neonatal outcomes

The processes of NR are dependent and follow a specified order for every newborn. A logistic regression model performed independently for each NR process showed that removing the wet cloth after drying the newborn (OR = 2.90, *p* = 0.035, CI = 1.08–4.23) and keeping the baby warm after stimulation (OR = 3.30, *p* = 0.018, CI = 1.22–8.88) were significantly associated with survival of the newborn. Newborns with meconium presence in airway were 66% more likely not to survive past 1 h after resuscitation (OR = 0.34, *p* = 0.042, CI = 0.12–0.96). Gestation age ≥ 37 weeks was significantly associated with increased survival at 1 h post NR (OR = 1.38, *p* = 0.007, CI = 1.10–1.75) (See Table 5).

**Table 5:** Relationship between the NR processes, newborn characteristics and the neonatal outcomes

NR process	OR	*P* - value	95% CI
Drying/stimulation (*n* = 138)			
Dried thoroughly	0.88	0.876	0.18–4.23
Wet cloth removed	2.90	0.035*	1.08–7.76
Baby kept warm	3.30	0.018*	1.22–8.88
Airway clearance (*n* = 123)			
Checked airway	5.72	0.225	0.34–95.68
Meconium present in airway	0.34	0.042*	0.12–0.96
Suctioning airway before stimulation in meconium presence (*n* = 57)	5.02	**0.051**	1.00–25.34
Head in neutral position	2.74	0.076	0.90–8.32
Bag and Mask Ventilation (*n* = 66)			
BMV within the Golden minute	1.11	0.843	0.38–3.24
Call for help (*n* = 30)	1.88	0.607	0.17–20.61
Correct mask for BMV (*n* = 30)	0.84	0.866	0.12–6.03
Chest movements on BMV (*n* = 30)	1.94	0.420	0.39–9.70
BMV within 30–50 b/min (*n* = 30)	1.27	0.757	0.28–5.87
HR checked at 1 min (*n* = 30)	2.08	0.429	0.34–12.72
Newborn characteristic			
Gestation age (weeks)	1.38	0.007*	1.10–1.75
Birth weight (grams)	1.00	0.448	0.99–1.00

*p ≤ 0.05 statistically significant

## Discussion

HCPs working with newborns must be well equipped with NR knowledge and skills in order to reduce neonatal mortality related to birth asphyxia. Our findings showed that most HCPs had undergone formal NR trainings over a year ago before this study. Supportive supervision was equally conducted for most HCPs by either the unit manager or ward in – charge at least over 6 months prior to this study. Absence of frequent monitoring of NR skills and refresher trainings every 6 months for HCPs make NR skills to decline rapidly over time as shown by a recent multi – country study on Helping Babies Breathe [27]. Elsewhere, there is evidence that HCPs with over two years of experience working in maternity unit had better NR skills compared to those with less in the same unit [15]. It is expected that those with more years of experience working in the unit help impart the same knowledge and skills to the new HCPs who join the service along the continuum.

Our findings revealed that HCPs were skilled in key critical NR steps in airway maintenance to initiate spontaneous breathing. This finding is reported in other studies conducted in other health facilities in the country [16, 17]. This could be explained by the availability of NR guidelines and action plans in NR, formal and/or informal trainings in NR and the necessary equipment to provide care in the hospital. However, a few newborns miss out on the most important step: being kept warm adequately and this could result in further neonatal deaths precipitated by hypothermia. Immediate essential newborn care after birth will reduce the neonatal morbidity and mortality that are most prevalent at this critical first hour after birth [6].

Bag and mask ventilation was initiated for all newborns who did not establish breathing after drying and airway clearance (with secretions). Our study finding show that HCPs clearly recognize the indication for bag and ventilation in newborns without or gasping respirations as recommended [2, 24]. However, BMV intervention was not initiated within the golden minute for most resuscitations after failed stimulation and airway clearance to initiate breathing as recommended by the national and international guidelines [14, 24]. When breathing is delayed, the window of opportunity to reverse the consequences of asphyxia (reductions in important blood pressure & cerebral blood flow and cardiac arrest) is small. Our finding showed that HCPs’ understanding of the importance of initiating BMV within the Golden minute after birth was poor. Evidence indicates that there is significant improvement in myocardial function and cerebral oxygenation when BMV is initiated within the Golden minute [2].

There is evidence that most resuscitated newborns will initiate spontaneous breathing after simple stimulation with very few going through the advanced NR steps involving chest compressions and drugs [8]. This implies that even in resource limited settings, many babies with birth asphyxia only require simple interventions to be able to initiate spontaneous breathing without difficulties. Good NR skills in drying/stimulating and airway clearance in cases of airway obstruction due to secretions and ensuring that the babies are kept warm may be all that is required to avert most of the neonatal deaths due to birth asphyxia.

Secretions obstruct the airway worsening the asphyxia. Our findings showed that newborns born with meconium – stained amniotic fluid in the airway who did not start breathing on their own had reduced chances of survival in the first hour after birth. Similarly, HCPs’ NR skills were poor in airway clearance in presence of meconium in babies who did not start breathing on their own. Healthcare providers dried/stimulated newborns before airway clearance in presence of meconium in non – breathing newborns contrary to both the international and national guidelines on the management of meconium presence in the airway [2, 14, 24]. Clearing airway in meconium presence in newborns not breathing should be done before drying the baby thoroughly as meconium inhaled into the lungs can cause breathing problems [24]. Other studies done in Kenya also demonstrated that meconium presence was a predictor of birth asphyxia and yet this study found that clearing meconium before stimulation was poorly performed by majority of health workers observed [16, 17, 28]. This is despite the most recent national updates on NR guidelines revised in 2013 being available [14]. This indicates that there is a knowledge deficit and/or lack of regular updates on the part of the HCPs on the significance of meconium in the airway. As a result, many newborns born through meconium may suffer both short term and long term sequelae ranging from birth asphyxia and eventual early neonatal death.

Bag and mask ventilation with room air and subsequent transfer to positive pressure ventilation using the nasal cannulae for unstable newborns was the standard practice on oxygen use during resuscitation. This is to ensure that asphyxiated newborns are given the highest quality of care to improve their chances of survival as recommended [24]. This is critical as in cases where there is no oxygen, HCPs may tend to give priority to sourcing for oxygen instead of starting ventilation using bag and mask with room air and may cause loss of critical seconds/minutes in the first hour after birth. This is similar from other global findings where only a small number of newborns (less than 1%) may fail to respond to the initial treatment and thus require advanced resuscitation [29].

Potentially harmful/inappropriate NR practices are still prevalent in the labor wards. This finding is widely reported in the most recent studies on adherence of HCPs to the national NR guidelines in Kenya [16, 17, 28, 30]. This can be explained by the fact that most HCPs had undergone NR trainings to improve their knowledge and skills more than a year ago before this study. Recent evidence in Helping Babies Breathe training in Kenya and India has shown that NR skills decline more than knowledge over time [27].

Prematurity is among the top three causes of neonatal mortality in Kenya [12] and the leading cause globally [3]. Resuscitated newborns delivered at gestation age ≥ 37 weeks had increased chances of survival in our study. Absence of sophisticated equipment and personnel for specialized intensive newborn care for preterm babies at the facility could explain this disparity. This include the recommended pulse oximetry for deciding on the need for supplemental oxygen and to monitor the needed concentration of oxygen related to its benefits on mortality [2]. Healthcare providers need the evidence – based training required for urgent specialized care for premature birthing mothers and their premature babies to improve the perinatal outcomes. Sophisticated equipment and personnel with the special skill set for caring for preterm babies at the regional referral facility will benefit clients seeking care at the regional referral facilities.

This study did not consider possible maternal factors that could have led to the deaths of the resuscitated newborns. The Hawthorn’s effect is a major drawback in direct observation studies; however, this helps to observe the ‘where’ and ‘when’ of the ongoing process/situation/behavior wanted. Multiple resuscitations per HCP were observed on the assumption that after a few minutes, the HCP would become accustomed to our presence and function in a more natural fashion. The small number of observations for the NR steps after failed initial stimulation and airway maintenance entails that our results should be interpreted in light of the small sample size.

## Conclusions

The hospital is prepared with the basic equipment to provide NR with nurses/midwives being the primary providers of NR services. In addition, training in neonatal resuscitation for HCPs is poorly spaced allowing deterioration of key skills. Nurses/midwives and other HCPs providing NR need regular cost – effective NR trainings with focus on maintaining the warm chain for the newborns, airway clearance in meconium – stained amniotic fluid in airways of newborns not breathing, bag and mask ventilation and care for premature babies focusing on the knowledge and practical skills at least after every 6 months. Besides, mentorship programs for the new HCPs in the maternity unit by the more experienced HCPs in the maternity can help to pass knowledge and skills to the new staff joining the unit [31].

## Additional file

Additional file 1: Structured observational checklist. (PDF 561 kb)

## Abbreviations

BEmONC: Basic Emergency Obstetric and Newborn Care
BMV: Bag and Mask Ventilation
CI: Confidence Interval
CPAP: Continuous Positive Airway Pressure
HBB: Helping Babies Breathe
HCP: Health care provider
NR: Neonatal Resuscitation
OR: Odds Ratio
RA: Research Assistant
WHO: World Health Organization
