# Supplementary material for: Practice and outcomes of neonatal resuscitation for newborns with birth asphyxia at Kakamega County General Hospital, Kenya: a direct observation study
Source: BMC Pediatr. 2018 May 15;18:167. doi: 10.1186/s12887-018-1127-6 (PMC5953400; doi:10.1186/s12887-018-1127-6)
Supplement: Supplementary file 1 — Structured observational checklist. (PDF 561 kb) [file 12887_2018_1127_MOESM1_ESM.pdf]

STRUCTURED OBSERVATIONAL CHECKLIST

SECTION A: HEALTHCARE PROVIDER BACKGRROUND (THIS SECTION REFERS TO THE HEALTHCARE PROVIDER WHO PERFORMED THE NEWBORN RESUSCITATION).
(*Please respond by ticking in the box next to the answer most suitable for you)*

PARTICIPANT CODE: _______________________ DATE: ________________

BIODATA

1. YOUR AGE (YEARS): _______________

2. GENDER:

MALE		FEMALE	

TRAINING AND QUALIFICATION

3. **HEALTH PROVIDER LEVEL**

1	Specialist (Obs/Gyn)	
2.	Medical Officer	
3.	Nursing Officer/Midwife	
4.	Clinical Officer	
5.	Other	

4. **HIGHEST QUALIFICATION ACHIEVED HEALTH PROVIDER LEVEL OF TRAINING**

1	Master’s Degree	
2.	Bachelor’s Degree	
3.	Diploma	
4.	Certificate	
5.	Other	

5. **HAVE YOU EVER ATTENDED A NEONATAL RESUSCITATION TRAINING?**
YES…….......... (PROCEED TO QUESTION 6)
NO……………. (PROCEED TO QUESTION 8)

6. **PRIOR NR TRAINING ATTENDED (TICK ALL THAT APPLY)**

1	Helping Babies Breath (HBB)	
2	Pediatric Advanced Life Support (PALS)	
3	Emergency Triage Assessment and Treatment (ETAT/ETAT +)	
4	European Pediatric Life Support (EPLS)	
5	Other (please specify)	

7. **DURATION (MONTHS/YEARS) SINCE LAST NR TRAINING (INCLUDE FOR ALL TRAININGS ATTENDED)**

YEARS……………………………..MONTHS……………………….

SUPERVISION AND EXPERIENCE

8. **HAVE YOU EVER HAD SUPPORT SUPERVISION IN NR IN THE WARD?**
   YES …………………………. (PROCEED TO QUESTION 9)
   NO…………………………… (PROCEED TO QUESTION 11)

9. **TIME SINCE MOST RECENT STAFF SUPERVISION**

1	< 6 months	
2	6 - 12 months	
3	> 12 months	

10. **WHO DID THE SUPERVISION?**

1	WARD IN – CHARGE	
2	UNIT MANAGER	
3	PUBLIC HEALTH NURSE	
4	WARD MATE/COLLEAGUE	

11. **DURATION OF PRACTICE IN MATERNITY UNIT (MONTHS/YEARS):**

    YEARS…………………………………MONTHS…………………………….

SECTION B: CLINICAL PROTOCOLS AND GUIDELINES (Tick where applicable)

This section to be completed by the principal researcher or research assistant

	JOB AIDS	YES	NO	REMARKS
1	Are visual NR action plans/guidelines present at the resuscitation areas?			
2	Do the healthcare workers refer to the NR guidelines/action plans during resuscitation?			
3	Are there flip charts for on job resuscitation training among healthcare providers in the labor unit?			

SECTION C: BASIC NEONATAL RESUSCITATION EQUIPMENT CHECKLIST

(To be filled in at the start of each resuscitation by the principal researcher or research assistant)
Instructions: Please tick in appropriate column to indicate if equipment is present or absent. The percentages will be calculated later.

STATION: LABOR WARD ___ MATERNITY THEATRE____NEWBORN UNIT____

PERMANENT ITEM	PRESENT	ABSENT	IN WORKING CONDITION
Warmer/ resuscitaire			
Oxygen source			
Suction machine			
Ambu bag (500mls)			
Clock			

TEMPORARY ITEM	PRESENT	ABSENT	REMARKS
Suction tube (6F,8F and 10F)			
Bulb suction device (penguin device, coloured bulb sucker)			
Face mask (preterm: size 0; term: size 1)			
Mode of oxygen delivery (nasal catheter, nasal prongs, face mask)			
Oxygen tubing			
Clean dry towels (2)			

SECTION D: NEWBORN RESUSCITATION OBSERVATION CHECKLIST

Instructions: *Please tick to the choice that applies to resuscitation being observed*

NO. OF OBSERVATIONS: …………. /5 DATE………………………….

SHIFT: DAY…….. EVENING….. … NIGHT………

STATION: LABOR WARD……..MATERNITY THEATRE……..NEWBORN UNIT…….

RESUSCITATION PROCEDURE (*please tick yes/ no depending on observation made during resuscitation)*

**PARAMETER**	**OBSERVATION**	**YES**	**NO**
**PREPARATION FOR NEWBORN RESUSCITATION**			
**A. PREPARATION FOR RESUSCITATION**	1. Prepares area for resuscitation		
	2. Checked availability of resuscitation equipment		
	3. Check equipment: Ventilation bag, Full term mask, Preterm mask, Suction bulb, Warmth (warmer/clothing).		
	4. Identify a helper		
Remarks			
**IMMEDIATELY AT BIRTH (FOR NEWBORN WHO DID NOT BREATHE AT BIRTH)**			
**B. DRYING/ STIMULATING**	1. Was the baby dried thoroughly? (by gently rubbing the back)		
	2. Was the wet cloth removed?		
	3. Was the baby kept warm?		
	Did the baby respond after drying/stimulating?		
	**OUTCOME**		
	**REMARKS**		
**IF BABY NOT BREATHING AND/OR CRYING AFTER DRYING AND STIMULATION**			
**C. CHECK/OPEN AIRWAY**	1. Looked into airway?		
	a. *Was meconium present?		
	b. If yes, was suctioning of airway done before drying/stimulating?		

	*Was the child breathing before suctioning?		
	2. Was the baby's airway cleared with a suction bulb if unresponsive?		
	3. Was the baby's head positioned in a neutral position?		
	*Did the baby respond after clearing the airway?		
	**OUTCOME**		
	**REMARKS**		
**IF BABY DOES NOT RESPOND TO INITIAL RESUSCITATION EFFORTS (POOR OR NO BREATHING/GASPING)**			
**D. BAG AND MASK VENTILATION**	1. Was bag – and – mask ventilation (BMV) initiated?		
	*Time BMV initiated after birth (seconds)		
	2. Was BMV initiated within the Golden minute (60s)?		
	*Did the baby respond after this initial BMV?		
	**IF BABY DID NOT RESPOND AFTER THE INITIAL BMV (ADVANCED BMV)**		
	1. Did the healthcare provider call for help?		
	2. Was the correct mask size used during BMV? (Covers nose, mouth and makes a tight seal)		
	3. Were there chest movements with each ventilation?		
	4. Was the ventilation rate within 30 – 50 per minute?		
	5. Was the baby's heart rate checked at 1 min?		
	Did BMV continue if baby still unresponsive?		
	*Did the baby respond after this assisted BMV?		
	**OUTCOME**		
	**REMARKS**		
**IF POOR OR NO BREATHING/GASPING AFTER BMV**			
**E. SUPPORT VENTILATION (IMPROVE**	1. Was 1 effective breath for every 3 compressions for 1 min done?		
	2. Did the baby require support breathing (supportive oxygen)?		

**VENTILATION)**	*Did the baby respond after the support ventilation?		
	**OUTCOME**		
	**REMARKS**		

NAME OF OBSERVER: ………………………………………………

SECTION E: NEONATAL PROGRESS MONITORING AND OUTCOME FORM

Gestational Age (weeks):…………………………….Birth weight (grams):…………………………….

Mode of delivery: SVD Assisted delivery Caesarean section Breech

	OUTCOME	PARAMETER	YES	NO
	**AT 1 MIN APGAR SCORE……..**	Did The Baby Cry/Breathe After Drying/Stimulating at 1 Min?		
		Did The Baby Respond To Clearing Of Airway?		
		Others (specify)		
	**AT 5 MIN APGAR SCORE……..**	A - Skin color pink?		
		G- Crying or active withdrawal?		
		A-Active motion?		
		R- Good cry?		
		Others (specify)		
	**AT 10 MIN APGAR SCORE…….**	A - Skin color pink?		
		G- Crying or active withdrawal?		
		A-Active motion?		
		R- Good cry?		
		Others (specify)		
	**AT 1 HOUR**	Baby breathing well with mother		

		Baby alive on oxygen therapy		
		Baby alive in special care area/unit		
		**Others (specify)**		

SECTION F: INFECTION PREVENTION PRACTICES

To be completed after completion of NR procedure by the principal researcher or research assistant.

	TASK	YES	NO
**1**	CLEANING: Ventilation bag and mask device disassembled (suction device if possible) while still wearing gloves.		
**2**	DECONTAMINATION: all parts soaked in a 0.5% chlorine solution for 10 minutes.		
**3**	All parts washed with soap and water and rinsed carefully with clean water to remove all soap.		
**4**	HLD: Parts boiled in water for 10 – 20 minutes OR soak in activated glutaraldehyde/cidex solution, then rinsed well with boiled water OR steam autoclave.		
**5**	Dry completely and keep clean until next use		
**6**	**REMARKS**		
